# Supplementary material for: Complexome profiling of the Chlamydomonas psb28 mutant reveals TEF5 as an early PSII assembly factor
Source: Plant Cell. 2025 Mar 17;37(6):koaf055. doi: 10.1093/plcell/koaf055 (PMC12164590; doi:10.1093/plcell/koaf055)
Supplement: koaf055_Supplementary_Data [file koaf055_supplementary_data.zip › psb28_interactive.zip!/plots/intensity/qualityPlots/peptides/ATP11_Cre10.g437050.t1.2.html]

### Cre10.g437050.t1.2

Trivial name: ATP11  
SeqCoverage [median]: 17.10  
Mass: 24.26 kDa  
  
 

| Sequence | MissedCleavage | Mass [Da] | wtIntensity | muIntensity | Score |
| --- | --- | --- | --- | --- | --- |
| GDIIHEK | 0 | 810.42 | 0.00 | 322.14 | 101.28 |
| KPASFDFGAVLR | 1 | 1306.70 | 0.00 | 597.96 | 43.54 |
| TALELTR | 0 | 802.45 | 0.00 | 718.40 | 86.79 |
| VGSVLSADDYK | 0 | 1152.57 | 0.00 | 218.01 | 103.70 |
|  |  |  | 0.0 | 1856.5 |  |
